# Prognostic value of glycolysis markers in pancreatic cancer: A systematic review and meta-analysis

**DOI:** 10.3389/fonc.2022.1004850

**Published:** 2022-09-12

**Authors:** Chengcheng Wang, Ruiyuan Xu, Jianlu Song, Yuan Chen, Xinpeng Yin, Rexiati Ruze, Qiang Xu

**Affiliations:** Department of General Surgery, State Key Laboratory of Complex Severe and Rare Diseases, Peking Union Medical College Hospital, Chinese Academy of Medical Sciences, Peking Union Medical College, Beijing, China

**Keywords:** pancreatic cancer, glycolysis, prognosis, therapeutic targets, meta-analysis

## Abstract

**Introduction:**

Previous studies have investigated the prognostic significance of glycolysis markers in pancreatic cancer; however, conclusions from these studies are still controversial.

**Methods:**

PubMed, Embase, and Web of Science were systematically searched to investigate the prognostic role of glycolysis markers in pancreatic cancer up to May 2022. Pooled hazard ratios (HRs) with 95% confidence intervals (CIs) related to overall survival (OS), disease free survival (DFS), recurrence-free survival (RFS), and distant metastasis-free survival (DMFS) were calculated using the STATA 12.0 software.

**Results:**

A total of 28 studies comprising 2010 patients were included in this meta-analysis. High expression of the five glycolysis markers was correlated with a poorer OS (HR = 1.72, 95% CI: 1.34-2.22), DFS (HR = 3.09, 95% CI: 1.91-5.01), RFS (HR = 1.73, 95% CI: 1.21-2.48) and DMFS (HR = 2.60, 95% CI: 1.09-6.20) in patients with pancreatic cancer. In subgroup analysis, it was shown that higher expression levels of the five glycolysis markers were related to a poorer OS in Asians (HR = 1.85, 95% CI: 1.46-2.35, *P* < 0.001) and Caucasians (HR = 1.97, 95% CI: 1.40-2.77, *P* < 0.001). Besides, analysis based on the expression levels of specific glycolysis markers demonstrated that higher expression levels of GLUT1 (HR = 2.11, 95% CI: 1.58-2.82, *P* < 0.001), MCT4 (HR = 2.26, 95% CI: 1.36-3.76, *P* = 0.002), and ENO1 (HR = 2.16, 95% CI: 1.28-3.66, *P* =0.004) were correlated with a poorer OS in patients with pancreatic cancer.

**Conclusions:**

High expression of the five glycolysis markers are associated with poorer OS, DFS, RFS and DMFS in patients with pancreatic cancer, indicating that the glycolysis markers could be potential prognostic predictors and therapeutic targets in pancreatic cancer.

## Introduction

Pancreatic cancer is the seventh leading cause of cancer-related death worldwide due to its poor prognosis with almost as many deaths (n=466,003) as cases (n=495,773) (1), which was estimated to become the second most common cause of cancer-related death by 2030 in the U.S (2). Pancreatic ductal adenocarcinoma (PDAC), the most common type of pancreatic cancer, is the deadliest malignancy with a 5-year survival rate of less than 8% (3). The worse clinical outcomes of PDAC are due to the lack of early typical symptoms and the highly aggressive biological characteristics, emphasizing the urgent need for searching promising prognostic markers for clinical practice.

Malignant proliferating cancer cells usually rewire energy metabolism manners to meet the requirements of rapid cell growth, division, invasion, and migration, which is an emerging hallmark of cancer known as “energy metabolism reprogramming” (4). It has been well known that tumor cells tend to increase their glucose uptake and lactate production even in the presence of ample oxygen, which is described as the “aerobic glycolysis” or “Warburg effect” (5). The enhancement of glycolysis has been demonstrated to play critical roles in pancreatic tumorigenesis and development (6, 7).

Previous studies have shown that multiple key glycolytic enzymes such as glucose transporter 1 (GLUT1), monocarboxylate transporter 4 (MCT4), hexokinase 2 (HK2), pyruvate kinase M2 (PKM2) and enolase 1 (ENO1) are usually overexpressed in tumors and play critical roles in glycolysis pathway (8). Transport of glucose across the plasma membrane is an important regulator of glucose metabolism and is mediated by glucose transporter (GLUT) family proteins. Increased rate of glucose uptake in malignant cells is associated with elevated expression of GLUTs, especially GLUT1 (9). Since enhanced glycolysis is associated with lactate production, tumor cells must eliminate excessive lactate out of the cell to prevent cellular acidification, which is achieved by the upregulation of MCT4, a proton-coupled lactate transporter (10). Besides cellular membrane transporters, several enzymes located in cytoplasmic fractions also contribute to glycolytic flux. As the most influential functional enzyme of glycolysis, overexpression of HK2 promotes glycolysis process by catalyzing phosphorylation of glucose to glucose-6-phosphate (11). PKM2 is the key rate-limiting enzyme involved in the final step of the glycolysis pathway and catalyzes the last irreversible step in glycolysis, the conversion of phosphoenolpyruvate to pyruvate, while ADP is phosphorylated to form ATP (12). Moreover, ENO1, also known as alpha-enolase, a critical glycolytic enzyme that dehydrates 2-phosphoglycerate to phosphoenolpyruvate, is present both in cell surface and cytoplasm (13). It has been well demonstrated that these glycolysis enzymes contribute to biological behaviors of cancer.

Although emerging evidence has focused on the correlation between various glycolysis markers and pancreatic cancer, the conclusions remain controversial. In terms of GLUT1, previous studies investigating the relationship between GLUT1 expression and clinical outcomes of patients with pancreatic cancer have yet yielded conflicting results. For example, some studies have demonstrated the independent prognostic role of GLUT1 in pancreatic cancer (14–19), while other investigators revealed that the expression of GLUT1 was not associated with clinicopathological characteristics and prognosis of pancreatic cancer (20, 21). Therefore, a comprehensive meta-analysis was performed to clarify the prognostic role of glycolysis markers GLUT1, MCT4, HK2, PKM2, and ENO1 in pancreatic cancer.

## Materials and methods

### Literature search

Eligible studies relevant to the association of glycolysis markers and prognosis of pancreatic cancer were systematically searched in databases, including PubMed, Embase, and Web of Science, dated until May 2022. The language was restricted to English. The retrieval strategy was listed as follow: (glucose transporter 1 OR GLUT1 OR monocarboxylate transporter 4 OR MCT4 OR hexokinase 2 OR HK2 OR pyruvate kinase M2 OR PKM2 OR Enolase 1 OR ENO1) AND (pancreatic OR pancreas) AND (cancer OR tumor OR tumor OR carcinoma OR adenocarcinoma OR neoplasia OR neoplasm) AND (prognosis OR prognostic OR prognoses OR survival OR outcome).

### Inclusion and exclusion criteria

All included studies need to meet the following inclusion criteria (1): including patients with pathologically diagnosed pancreatic cancer; (2) glycolysis markers were measured using immunohistochemistry (IHC); (3) studies describing the correlation between the expression of glycolysis markers and survival outcome; (4) hazard ratio (HR) values and corresponding 95% confidence intervals (CIs) for overall survival (OS), disease free survival (DFS), recurrence-free survival (RFS) or distant metastasis-free survival (DMFS) were either reported or calculatable with the published data in studies. Exclusion criteria were as follows: (1) duplicated studies; (2) studies not based on humans subjects; (3) reviews, case reports, meta-analysis, and clinical trials; (4) studies without available data.

### Data extraction

The following information was extracted from each study: first author’s name, publication year, country, ethnicity, types of glycolysis markers, sample size, gender, follow-up duration, cancer subtype, detection method, cut-off values of glycolysis markers and outcome. If HRs and 95% CIs were provided in both univariate and multivariate analyses, the latter was adopted. If a study only had Kaplan–Meier survival curves, survival data was extracted from the curves by Engauge Digitizer software.

### Quality assessment

Newcastle-Ottawa Scale (NOS) was applied to comprehensively evaluate the quality of the included studies (22). NOS contained three domains: patient selection (0-4 points), comparability (0-2 points), and outcome (0-3 points). NOS scores ranged from 0-9 points. The studies with an NOS score of six or above were of high quality.

### Statistical analysis

The association of glycolysis markers with OS, DFS, RFS and DMFS was evaluated by pooled HRs and 95% CIs. The pooled odds ratios (OR) and 95% CIs were used to investigate the association between the expression of glycolysis markers with the clinicopathologic characteristics of pancreatic cancer. Heterogeneity among various studies was evaluated using *χ* ^2^ test and I^2^ statistic. The random-effects model was used when significant heterogeneity was detected (*P* < 0.1 or *I* ^2^ > 50%); otherwise, the fixed-effects model was applied. Subgroup analysis was performed to explore the potential causes of heterogeneity. Publication bias was estimated using Begg’s funnel plot and Egger’s test. All statistics were analyzed using Stata software version 12.0 (STATA Corporation, College Station, TX, USA). *P* < 0.05 was considered statistically significant.

## Results

### Characteristics of included studies

**Figure 1:**
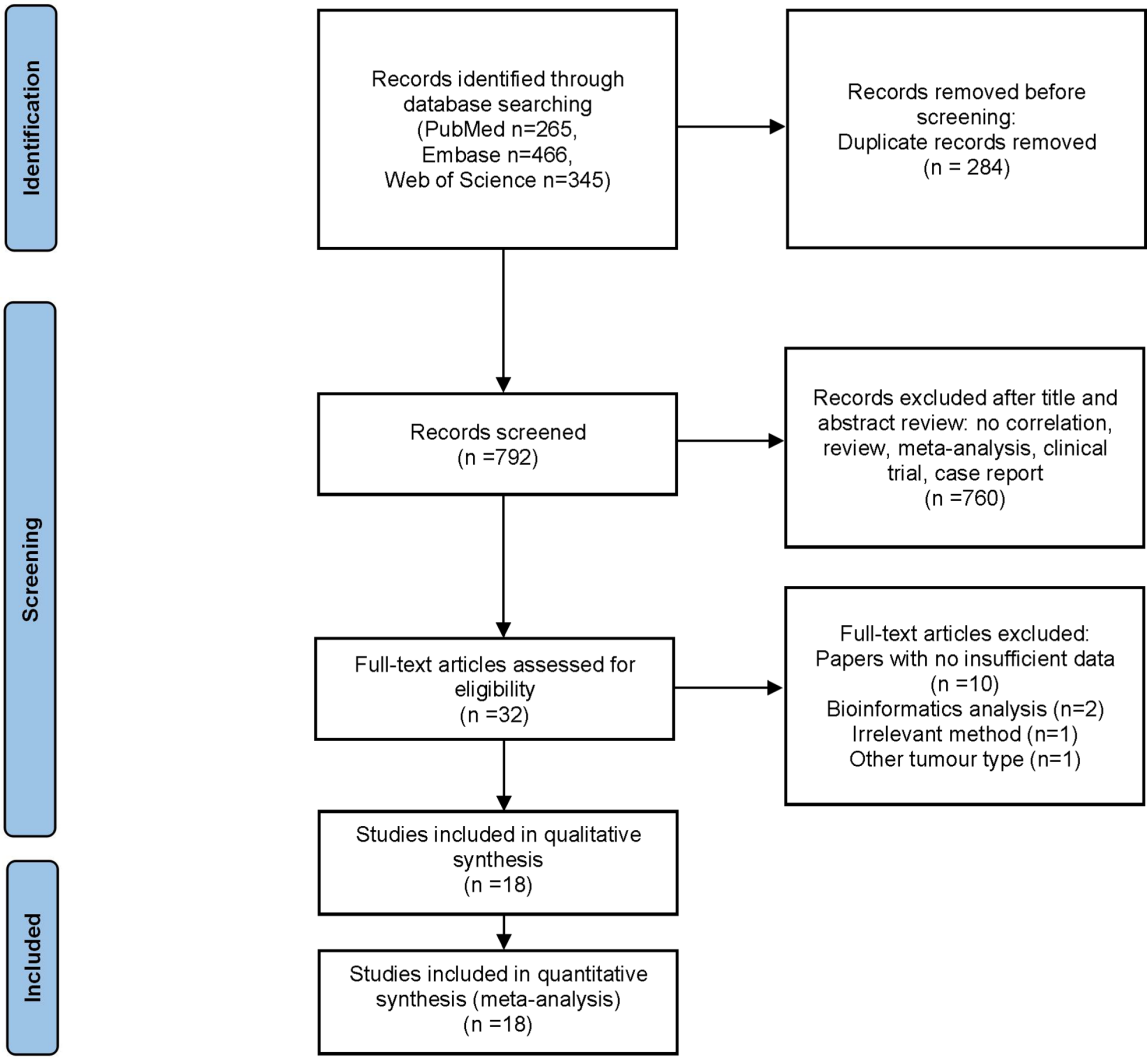
Flow diagram of study inclusion.

The flow diagram for study selection is illustrated in **Figure 1**. The characteristics of included studies are listed in **Table 1**. The initial literature search retrieved a total of 1076 publications from three databases. Among them, 265 articles came from PubMed, 466 from Embase, and 345 from Web of Science. After removing duplicates and manually screening titles and abstracts, the full text of the remaining 32 studies were assessed for eligibility. Fourteen articles were subsequently excluded for the following reasons: 10 papers were excluded due to data insufficiency, two were bioinformatic analysis, one used irrelevant method, and one was about other tumor. Finally, a total of 18 studies published between the year 2007 to 2020 were included in this meta-analysis (14–21, 23–32), made up of 28 studies and 2010 patients. The quality of the included studies was assessed using NOS, with all scoring of six or above (**Supplementary Table 1**). Majority of these studies assessed GLUT1 (n=11), whereas the rest assessed PKM2 (n=9), HK2 (n=5), ENO1 (n=2) and MCT4 (n=1). The sample size varied from 36 to 223 patients. In all eligible studies, the expression of glycolysis markers was detected using IHC. HR values and 95% CIs were directly provided in 8 studies, while HRs and 95% CIs of other studies were extracted using Engauge Digitizer 4.1 software with reported survival curves. The prognostic value of specific glycolysis markers was investigated by evaluating the overall survival (OS) in 20 studies, disease-free survival (DFS) in 2 studies, recurrence-free survival (RFS) in 4 studies, and distant metastasis-free survival (DMFS) in 2 studies.

**Table 1:** Characteristics of included studies.

Author year	Country	Ethnicity	Glycolysis marker	Sample size	Gender (M/F)	Follow-up (months)	Pathology	Detection method	Cut-off value	Outcome	HR [95% CI]	NOS score
Yang 2016 (20)	China	Asian	GLUT1	50	34/16	17 (6–35)	PC	IHC	Score≥9 (0-12)	OS	2.71 (0.93-7.91)	8
Yang 2016 (20)	China	Asian	HK2	50	34/16	17 (6-35)	PC	IHC	Score≥4 (0-12)	OS	0.94 (0.34-2.56)	8
Takahashi 2020 (14)	Japan	Asian	GLUT1	101	59/42	NA	PDAC	IHC	NA	OS DFS	OS: 3.68 (1.76-7.67) DFS: 3.53 (1.99-6.26)	6
Boira 2020 (15)	Spain	Caucasian	GLUT1	39	23/16	16 (9.7–39.2)	PDAC	IHC	Positive cells ≥ 80%	OS DFS	OS: 1.52 (0.57-4.1) DFS: 2.23 (0.91-5.47)	7
Lu 2016 (16)	China	Asian	GLUT1	53	29/24	NA	PC	IHC	Score≥2 (0-4)	OS	2.57 (0.46-14.32)	7
Lyshchik 2007 (21)	Japan	Asian	GLUT1	74	27/47	NA	PC	IHC	index ≥3	OS	1.52 (0.86-2.7)	7
Lyshchik 2007 (21)	Japan	Asian	HK2	74	27/47	NA	PC	IHC	index ≥ 3	OS	0.64 (0.34-1.18)	7
Pizzi 2009 (17)	Italy	Caucasian	GLUT1	60	30/30	NA	PDAC	IHC	Score≥3 (1–4)	OS	2.81 (1.1-8.0)	7
Chikamoto 2017 (18)	Japan	Asian	GLUT1	138	76/62	27	PC	IHC	Score≥11% (0-100%)	OS RFS	OS: 1.97 (1.140-3.488) RFS: 1.89 (1.18-3.01)	8
Kitasato 2014 (19)	Japan	Asian	GLUT1	41	21/20	NA	PDAC	IHC	Intensity score≥moderate	OS	1.86 (0.43-8.11)	7
Baek 2014 (23)	USA	Caucasian	MCT4	223	121/102	NA	PDAC	IHC	Score≥6 (0-9)	OS	2.264 (1.365-3.756)	7
Ogawa 2015 (24)	Japan	Asian	HK2	36	21/15	NA	PDAC	IHC	Score≥5 (0-10)	OS LRFS DMFS	OS: 2.57 (0.89-8.39) LRFS: 2.41 (0.69-8.46) DMFS: 2.41 (0.74-7.86)	7
Ogawa 2015 (24)	Japan	Asian	PKM2	36	21/15	NA	PDAC	IHC	Score≥5 (0-10)	OS LRFS DMFS	OS: 2.16 (0.82-6.1) LRFS: 2.22 (0.71-6.97) DMFS: 2.84 (0.79-10.29)	7
Mohammad 2016 (25)	UK	Caucasian	PKM2	72	39/33	NA	PC	IHC	Score≥4 (1-9)	OS	1.55 (0.84-2.86)	7
Calabretta 2016 (26)	Italy	Caucasian	PKM2	42	22/20	NA	PDAC	IHC	Score≥4 (0-5)	RFS	1.12 (1-4.4)	7
Xu 2017 (27)	China	Asian	PKM2	60	37/23	NA	PC	IHC	≥50%	OS	1.97 (1-3.89)	7
Li 2016 (28)	China	Asian	PKM2	90	57/33	NA	PDAC	IHC	Intensity score≥moderate	OS	2.21 (0.93-5.23)	6
Lockney 2015 (29)	USA	African American, Caucasian	PKM2	115	63/52	NA	PDAC	IHC	Score≥3 (0-12)	OS	0.57 (0.36-0.91)	7
Hu 2020 (30)	China	Asian	PKM2	77	51/26	NA	PDAC	IHC	Score≥6 (0-12)	OS	2.117 (1.309-3.426)	7
Wang 2019 (31)	China	Asian	ENO1	57	NA	NA	PDAC	IHC	Score≥6 (0-16)	OS	1.44 (0.50-4.18)	7
Sun 2017 (32)	China	Asian	ENO1	100	NA	NA	PC	IHC	Score≥4 (0-9)	OS	2.469 (1.348-4.522)	7

M/F, male/female; HR, hazard ratio; CI, confidence interval; IHC, immunohistochemistry; OS, overall survival, DFS, disease free survival, RFS, recurrence-free survival; DMFS, distant metastasis- free survival; NOS, Newcastle–Ottawa Scale; NA, not available.

**Figure 2:**
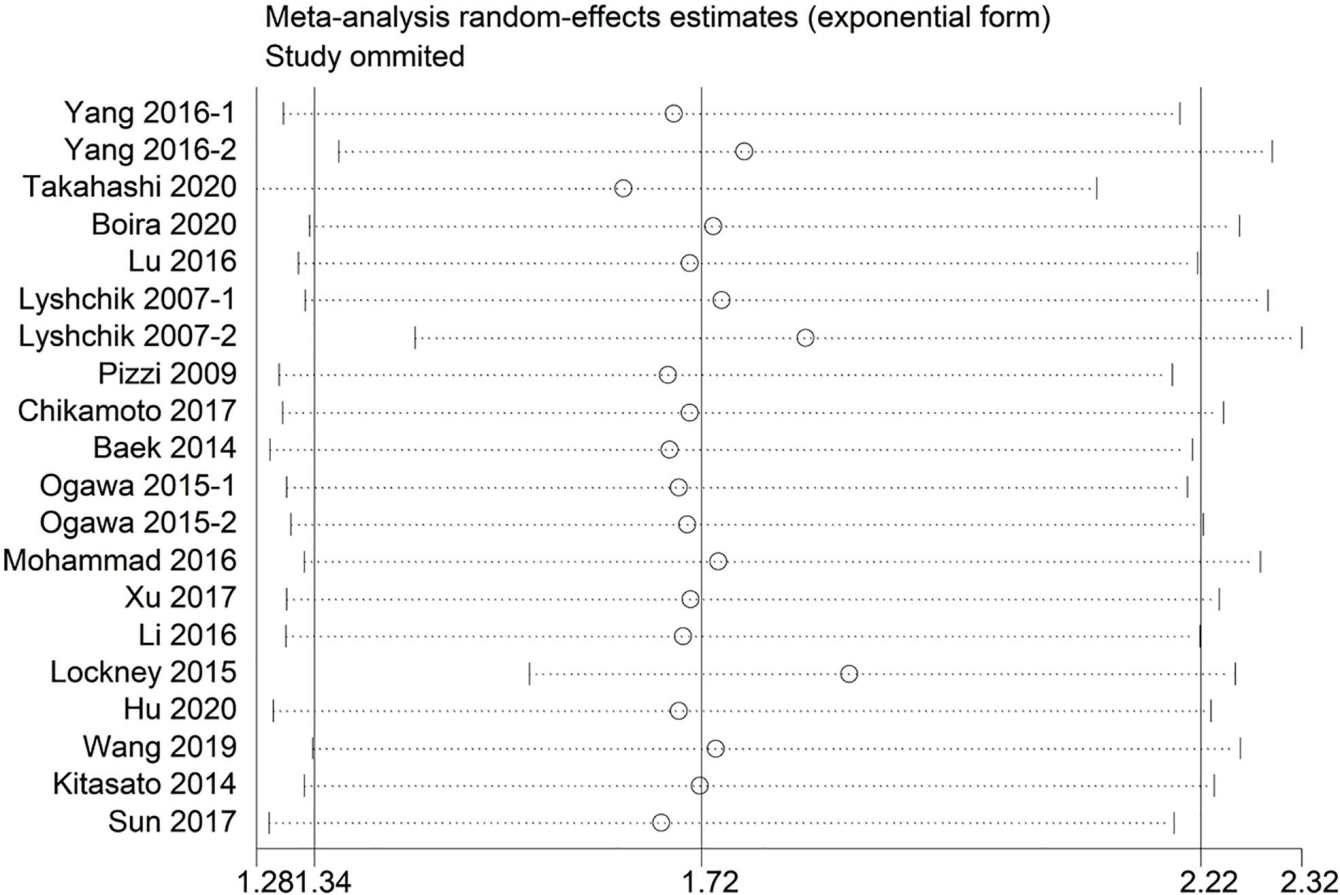
Sensitivity analysis between the expression levels of glycolysis markers and overall survival.

**Figure 3:**
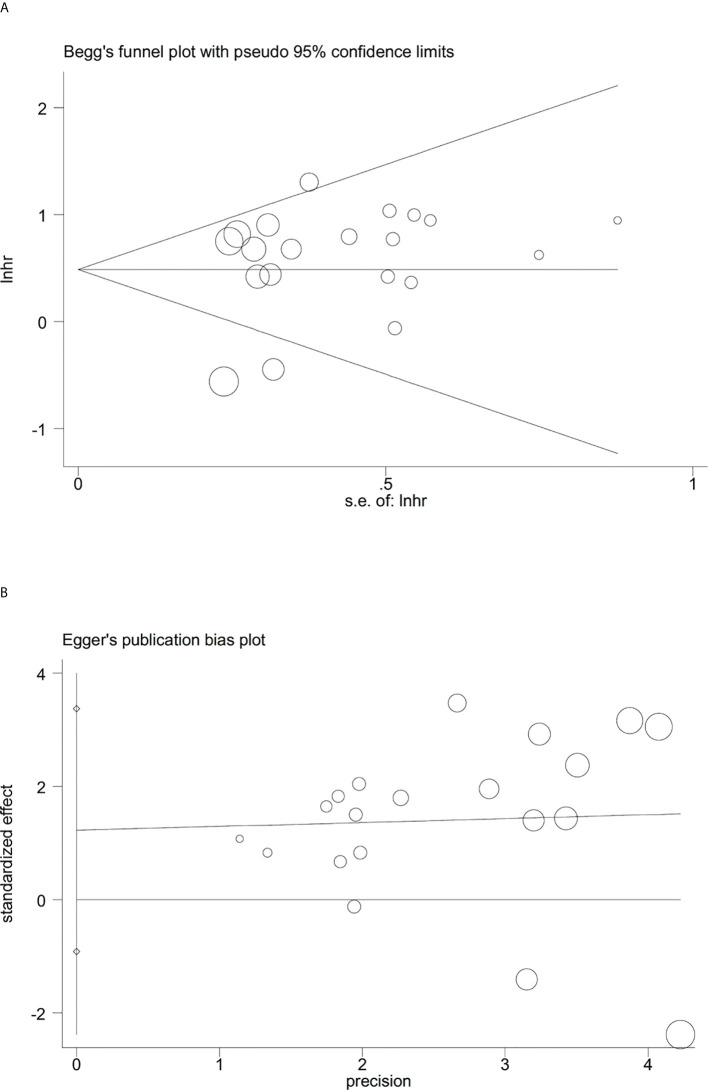
Evaluation of publication bias between the expression levels of glycolysis markers and overall survival. **(A)** Begg’s funnel plot, *P*=0.871; **(B)** Egger’s test, *P*=0.245.

Sensitivity analysis indicated that no significant heterogeneity was present among the included studies (**Figure 2**). Then, the publication bias for OS was evaluated, where neither Begg’s test (**Figure 3A**) nor Egger’s test (**Figure 3B**) showed a significant publication bias (*P*=0.871 and *P*=0.245, respectively).

### Glycolysis markers and OS in pancreatic cancer

**Figure 4:**
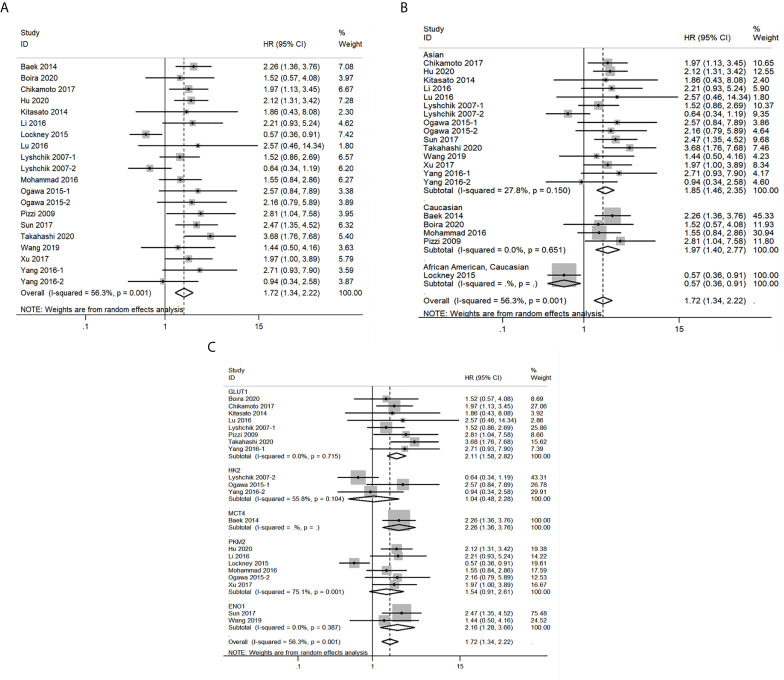
Forest plots of pooled hazard ratio (HR) for the association between the expression levels of glycolysis markers and overall survival of pancreatic cancer patients. **(A)** The overall group; **(B)** Subgroup analysis for ethnicity; **(C)** Subgroup analysis for glycolysis markers types. A random-effects model was applied.

Next, the relationship between expression levels of the 5 glycolysis markers and OS in pancreatic cancer from 20 studies that included 1546 patients was evaluated. As described in **Figure 4A**, results indicated that a higher expression of glycolysis markers correlated with poor OS of pancreatic cancer (HR = 1.72, 95% CI: 1.34-2.22). Owing to a significant heterogeneity among studies (I^2^ = 56.3%, *P*=0.001), a random-effects model was applied. Then, subgroup analysis was applied to explore causes of heterogeneity. The regional subgroup analysis indicated that high expression levels of the five glycolysis markers correlated with poorer OS in Asians (HR = 1.85, 95% CI: 1.46-2.35, *P* < 0.001) and Caucasians (HR = 1.97, 95% CI: 1.40-2.77, *P* < 0.001) (**Figure 4B**). The decreased heterogeneity from 56.3% to 0%-27.8% following the subgroup analysis indicated that the differences in regions are the main cause of heterogeneity. In addition, it was shown that higher expression levels of GLUT1 (HR = 2.11, 95% CI: 1.58-2.82, *P* < 0.001), MCT4 (HR = 2.26, 95% CI: 1.36-3.76, *P* = 0.002), and ENO1 (HR = 2.16, 95% CI: 1.28-3.66, *P* =0.004) correlated with poorer OS in pancreatic cancer in the subgroup analysis based on the expression levels of specific glycolysis markers (**Figure 4C**). The various heterogeneity in different subgroups (GLUT1: I^2 ^= 0%, HK2: I^2 ^= 55.8%, PKM2: I^2 ^= 75.1% and ENO1: I^2 ^= 0%) suggested that different glycolysis markers may not be the main cause of heterogeneity.

### Glycolysis markers and DFS in pancreatic cancer

**Figure 5:**
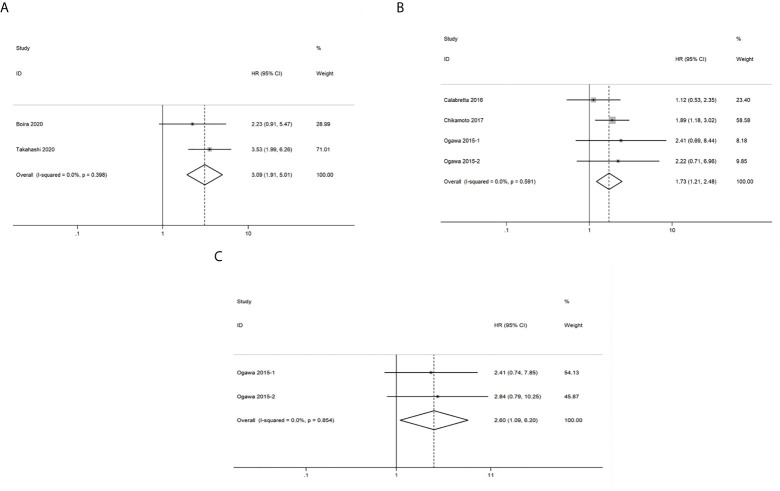
Forest plots of pooled hazard ratio (HR) for the association between the expression levels of glycolysis markers and **(A)** Disease free survival **(B)** Recurrence-free survival **(C)** Distant metastasis-free survival of pancreatic cancer patients. A fixed-effects model was used.

A total of 2 studies related to GLUT1 that included 140 patients were included in the pooled survival analysis of DFS. As shown in **Figure 5A**, results indicated that GLUT1 overexpression predicted a poor DFS of patients with pancreatic cancer (HR = 3.09, 95% CI: 1.91-5.01). A fixed-effects model was used since no significant heterogeneity exists between the 2 studies (I^2 ^= 0%, *P*=0.398). However, subgroup analysis was not performed as the number of included studies was too small.

### Glycolysis markers and RFS in pancreatic cancer

A total of 252 patients from 4 studies were evaluated to examine the correlation between expression levels of GLUT1, HK2 and PKM2 and RFS, and the meta-analysis of these studies showed that upregulation of the three glycolysis markers indicated a poor RFS of pancreatic cancer (HR = 1.73, 95% CI: 1.21-2.48) (**Figure 5B**). There was no significant heterogeneity between the studies (I^2 ^= 0%, *P*=0.591), therefore, a fixed-effects model was used to calculate the pooled HRs. Likewise, subgroup analysis was not possible due to the limited number of studies that reported RFS.

### Glycolysis markers and DMFS in pancreatic cancer

We then analyzed the data from 2 studies with 72 patients to determine the relationship between DMFS and the expression levels of HK2 and PKM2 in pancreatic cancer. We observed that significant correlation between expression levels of the two glycolysis markers and DMFS of patients with pancreatic cancer (HR = 2.60, 95% CI: 1.09-6.20) (**Figure 5C**). Moreover, there was no significant heterogeneity between the studies (I^2 ^= 0%, *P*=0.854). Similarly, due to the limited number of included studies, subgroup analysis was not available.

### Glycolysis markers and clinicopathologic characteristics of pancreatic cancer

The relationship between GLUT1 and PKM2 and clinicopathologic characteristics of pancreatic cancer was further analyzed. In terms of GLUT1, a total of 9 features from 8 studies including gender, age, tumor location, tumor differentiation, TNM stage, lymph node metastasis, perineural invasion, vascular invasion and resection margin were investigated in **Table 2** and **Supplementary Figure 1**. However, the combined data indicated that the correlations between the expression of GLUT1 and the clinical characteristics were not significant. Moreover, we analyzed a total of 7 features from 9 studies that reported the association between the expression of PKM2 and the clinicopathologic features including gender, tumor location, tumor differentiation, TNM stage, lymph node metastasis, perineural invasion and resection margin (**Table 3** and **Supplementary Figure 2**). Similarly, no statistical correlations were observed between PKM2 expression and the clinicopathologic characteristics of pancreatic cancer.

**Table 2:** The relationship between GLUT1 and clinicopathologic characteristics.

Features	No. of studies	Test for association			Test for heterogeneity		
		OR	95% CI	*P*	Chi^2^	I^2^ (%)	*P*
Gender (Male vs. Female)	5	1.03	0.72-1.48	0.875	2.41	0	0.661
Age (<60 years vs. >60 years)	2	1.03	0.49-2.18	0.941	0	0	0.947
Tumor location (Head vs. Body and tail of pancreas)	3	1.17	0.73-1.86	0.519	0.15	0	0.927
Tumor differentiation (poor vs. well/moderate)	5	1.20	0.82-1.75	0.359	1.15	0	0.887
TNM stage (III + IV vs. I + II)	2	1.47	0.66-3.26	0.341	0.07	0	0.799
Lymph node metastasis (Present vs. Absent)	4	1.13	0.76-1.67	0.543	1.45	0	0.695
Perineural invasion (Present vs. Absent)	3	1.03	0.53-1.98	0.939	0.51	0	0.775
Vascular invasion (Present vs. Absent)	3	0.87	0.49-1.54	0.633	0.58	0	0.749
Resection margin (R1 vs. R0)	2	1.06	0.52-2.17	0.879	0.04	0	0.841

**Table 3:** The relationship between PKM2 and clinicopathologic characteristics.

Features	No. of studies	Test for association			Test for heterogeneity		
		OR	95% CI	*P*	Chi^2^	I^2^ (%)	*P*
Gender (Male vs. Female)	6	1.01	0.72-1.41	0.975	1.21	0	0.944
Tumor location (Head and neck vs. Body and tail of pancreas)	3	0.97	0.61-1.53	0.896	1.59	0	0.452
Tumor differentiation (poor vs. well/moderate)	6	1.23	0.87-1.74	0.247	4.82	0	0.438
TNM stage (II +III + IV vs. I)	4	1.62	0.97-2.70	0.063	4.12	27.3	0.248
Lymph node metastasis (Present vs. Absent)	5	1.34	0.81-2.20	0.252	0.63	0	0.960
Perineural invasion (Present vs. Absent)	4	1.03	0.68-1.56	0.889	2.01	0	0.570
Resection margin (R1 vs. R0)	2	0.76	0.42-1.38	0.368	0.12	0	0.729

## Discussion

Energy metabolism reprogramming including aberrant glucose, amino acid, lipid metabolism and other bioenergetic metabolism pathways confers tumor cells the ability to acquire necessary nutrients and biomass in order to survive and proliferate under a frequently severe and nutrient-limited microenvironment (33, 34). Among various metabolic manners, aerobic glycolysis which is the central pathway of dysregulation of glucose metabolism has been well demonstrated in many tumors. Notably, upregulation of key enzymes including GLUT1, HK2, phosphofructokinase (PFK), TP53-induced glycolysis and apoptosis regulator (TIGAR), PKM2, ENO1, lactate dehydrogenase A (LDHA) and MCT4 in glycolytic pathway contributes to the increased glycolytic flux and promotion of malignant behaviors of various cancers including pancreatic cancer (7, 35). In addition, therapeutic strategies for targeting glycolytic enzymes are emerging as promising anti-cancer candidates. 3-bromopyruvate (3-BrPA), an inhibitor of HK2, has been well proved to block the growth and progression of pancreatic cancer both *in vitro* and *in vivo* (36, 37). In addition to HK2, the other glycolytic rate-limiting enzyme PFK1 could be allosterically activated by fructose-2,6-bisphosphate (F2,6BP) mediated by well-established glucose metabolism regulators 6-phosphofructo-2-kinase/fructose-2,6-bisphosphatases (PFKFBs) (38). PFK15, a PFKFB3 inhibitor, has been demonstrated to reduce glycolysis and plasma membrane calcium ATPases (PMCAs) activity, resulting in calcium overload and cell apoptosis in PDAC (39). Treating pancreatic cancer xenografts with FX11 which is a small-molecule inhibitor of LDHA suppressed tumor progression (40). Additionally, the combination of LDHA inhibitors and gemcitabine displayed synergistic cytotoxic activity against pancreatic cancer cells under hypoxic conditions (41). In terms of clinical applications, in a phase I clinical trial in patients with advanced solid tumors, including pancreatic cancer, the glycolytic inhibitor 2-deoxy-D-glucose (2DG) in combination with chemotherapeutic agent docetaxel produced tolerable adverse effects and feasible clinical benefit (NCT00096707) (42). Given the potential benefits for targeting glycolytic enzymes, more clinical trials of specific glycolysis inhibitors in patients with pancreatic cancer should be considered.

In the present meta-analysis, we analyzed data from 28 studies consisting of 2010 patients to determine the prognostic value of five glycolysis markers in pancreatic cancer. The results showed that high expression levels of glycolysis markers significantly correlated with worse OS, DFS, RFS and DMFS. The conclusion is consistent with the results of most studies included in this meta-analysis. In terms of subgroup analysis for OS, analysis by ethnicity showed that higher expression of glycolysis markers was correlated with shorter OS in both Asians and Caucasians. This implies that malignant glycolysis phenotypes of pancreatic cancer are common phenomenon in humans. Moreover, subgroup analysis of the expression levels of specific glycolysis markers highlighted that higher expression levels of GLUT1, MCT4, and ENO1 correlated with poorer OS in pancreatic cancer patients, while the expression levels of HK2 and PKM2 did not. These results indicated that the glycolysis pathways of pancreatic cancer may vary in diverse glycolysis markers and GLUT1, MCT4, and ENO1 may be potential therapeutic targets in treating pancreatic cancer. Due to the limited number of included studies, subgroup analysis for DFS, RFS and DMFS in pancreatic cancer patients was not performed in the present meta-analysis. Hence, more studies examining the correlation between expression levels of glycolysis markers and DFS, RFS and DMFS of pancreatic cancer patients need to be conducted. Moreover, we also analyzed the association between GLUT1 and PKM2 and clinicopathologic features including gender, age, tumor location, tumor differentiation, TNM stage, lymph node metastasis, perineural invasion, vascular invasion, and resection margin in pancreatic cancer. However, no significant correlations were observed. The reason may be attributed to limited number of studies and samples and the high levels of heterogeneity between various studies. Therefore, further studies are warranted to evaluate the role of glycolysis markers in clinicopathologic characteristics of pancreatic cancer patients in the future.

In recent years, ^18^F-fluorodeoxyglucose positron emission tomography (FDG-PET) based on the increased glucose metabolism of malignant cells has been being used as a valuable functional imaging technology in clinical practice for various malignancies. Altered expression levels of GLUT has been demonstrated to be associated with enhanced FDG uptake quantified by standardized uptake values (SUVs) in tumor cells (43). Interestingly, previous studies investigating the association between the expression of GLUT1 and SUVs in pancreatic cancer have yielded conflicting results. Chikamoto et al. suggested that a significant relationship existed between high maximum standardized uptake value (SUV_max_) and the expression of GLUT1 (18), whereas other studies indicated that GLUT1 expression was not significantly correlated with SUV_max_ (14, 20). Similarly, a recent meta-analysis suggested that only a moderate association was identified between the expression of GLUT1 and SUVs derived from FDG-PET in multiple tumor types (44). Therefore, it can be deduced that the correlations between glucose hypermetabolism *in vivo* displayed by FDG-PET and enhanced glycolytic phenotype of tumor cells are more complex than the single expression of GLUT1. Other key proteins of the glucose metabolism such as other members of GLUT family and HK2 might contribute to the alterations in SUVs, which needs to be confirmed in further studies.

The pancreatic tumor microenvironment (TME) consisting of cancer cells, stromal cells, and extracellular components is considered to play a pivotal role in multiple malignant biological behaviors including carcinogenesis, proliferation, invasion, metastasis, angiogenesis, immunosuppression and chemoresistance. It should be noted that stromal cells could be induced by cancer cells to present a glycolytic phenotype and support mitochondrial oxidative phosphorylation (OXPHOS) in cancer cells to fuel cell proliferation and growth, which was described as “the reverse Warburg effect” (45). As a key component in pancreatic TME, pancreatic stellate cells (PSCs) could be induced a shift to glycolysis in Caveolin-1-ROS positive feedback signaling, presenting upregulated expression of glycolytic enzymes HK2, 6-phosphofructokinase (PFKP), PKM2 and GLUT1 with downregulated expression of OXPHOS enzymes (46). Cancer-associated fibroblasts (CAFs), which develops mostly from activated PSCs, showed elevated expression of lactate dehydrogenase (LDHA), PKM2 and enhanced glucose uptake capability and lactate production (47, 48). The enhanced glycolysis of both PSCs and CAFs contribute to the proliferative and invasive capacities of pancreatic cancer cells. Moreover, pancreatic cancer is characterized by extensive infiltration of immunosuppressive cells such as regulatory T cells (Tregs), tumor-associated macrophages (TAMs) and myeloid-derived suppressor cells (MDSCs). It has been well demonstrated that glycolytic by-product lactic acid not only blunts tumor immunosurveillance by T and NK cells but also enhances immunosuppressive phenotype of Tregs, TAMs and MDSCs (49–52). A recent study further indicated that a terminally differentiated subpopulation of tumor-associated neutrophils exhibited hyperactivated glycolytic activity in PDAC TME promotes pro-tumor and immunosuppression functions of neutrophils (53). Therefore, targeting enhanced glycolysis of multiple components in the TME, not just tumor cells, may contribute to the development of novel therapeutic strategies against PDAC.

Several limitations should be addressed for this meta-analysis. First, given the current lack of a universal cut-off value for various glycolysis markers, different cut-off values were applied in different included studies. Second, some of the HRs and 95% CIs were estimated by extracting data from the survival curves, which might produce statistical deviations. Third, many studies included did not provide follow-up durations and they may have contributed to the bias. Fourth, the sample size of some studies is small. Considering the limitations of the present study, additional high-quality, large-scale, long-term studies need to be conducted.

## Conclusions

This meta-analysis demonstrated that high expression of the five glycolysis markers correlated with poorer OS, DFS, RFS and DMFS in pancreatic cancer patients, suggesting that glycolytic pathway enzymes are potential prognostic biomarkers and promising therapeutic targets in patients with pancreatic cancer. However, due to the limitations of this study, more high-quality and well-designed studies need to be performed in the future.

## Data availability statement

The original contributions presented in the study are included in the article/**Supplementary Material**. Further inquiries can be directed to the corresponding author.

## Author contributions

QX, CW, and RX conceived and designed the study. CW, RX, and JS drafted the manuscript, acquired the data, and performed the meta-analysis. YC, XY, and RR revised the manuscript for important intellectual contents. All authors read and approved the final manuscript.

## Funding

This study was supported by the CAMS Innovation Fund for Medical Sciences (2021-1-I2M-002), National Natural Science Foundation of China (82102810 and 81970763) and fellowship of China Postdoctoral Science Foundation (2022T150067).

## Conflict of interest

The authors declare that the research was conducted in the absence of any commercial or financial relationships that could be construed as a potential conflict of interest.

## Publisher’s note

All claims expressed in this article are solely those of the authors and do not necessarily represent those of their affiliated organizations, or those of the publisher, the editors and the reviewers. Any product that may be evaluated in this article, or claim that may be made by its manufacturer, is not guaranteed or endorsed by the publisher.
