# Supplementary material for: Prognostic value of glycolysis markers in pancreatic cancer: A systematic review and meta-analysis
Source: Front Oncol. 2022 Sep 12;12:1004850. doi: 10.3389/fonc.2022.1004850 (PMC9510923; doi:10.3389/fonc.2022.1004850)
Supplement: Supplementary file 1 [file DataSheet_1.docx]

**Supplementary material**

**
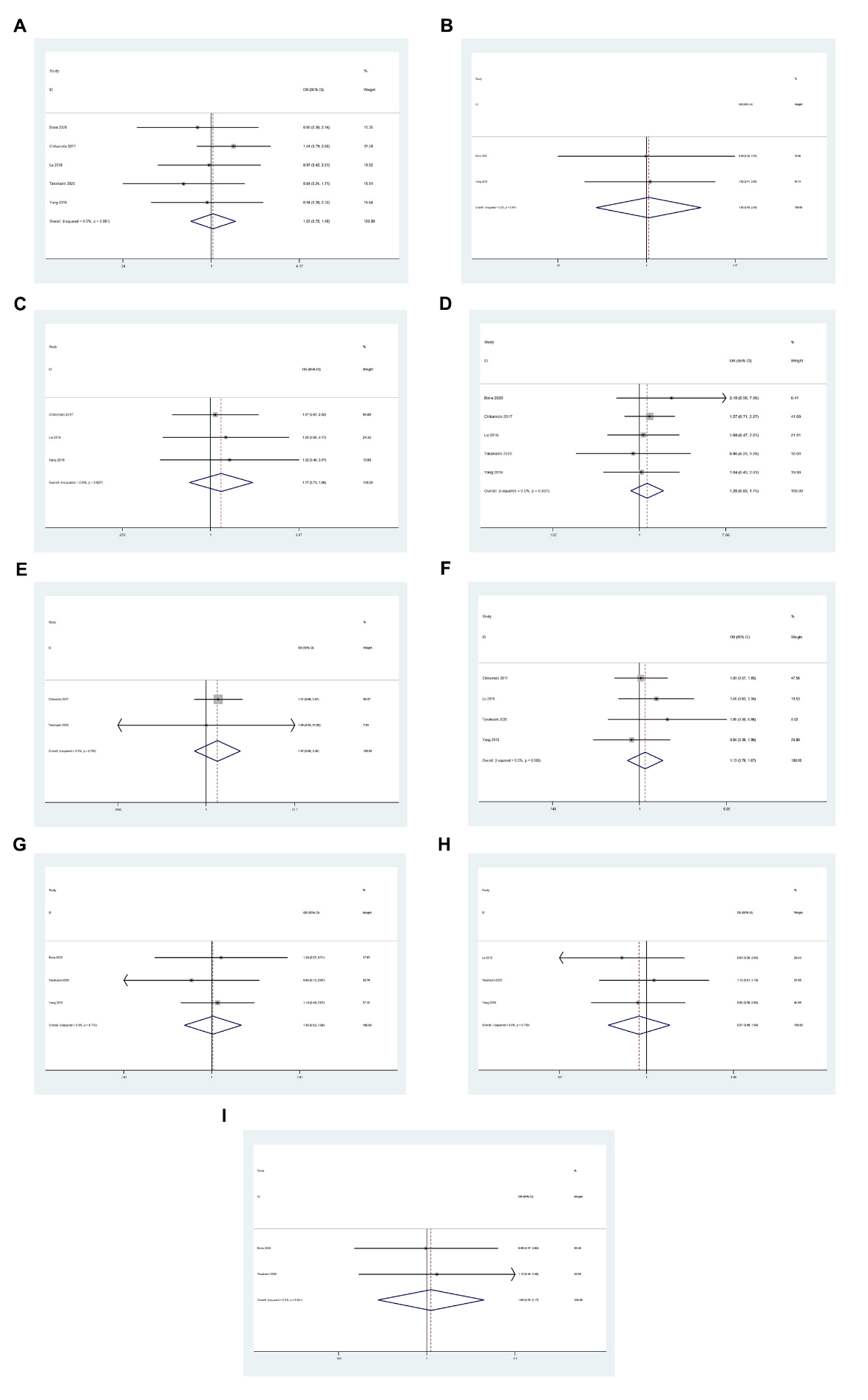
**

Supplementary Figure 1. Forest plots for the relationship between GLUT1 and clinicopathologic characteristics in pancreatic cancer. (A) Gender; (B) Age; (C) Tumor location; (D) Tumor differentiation; (E) TNM stage; (F) Lymph node metastasis; (G) Perineural invasion; (H) Vascular invasion; (I) Resection margin.


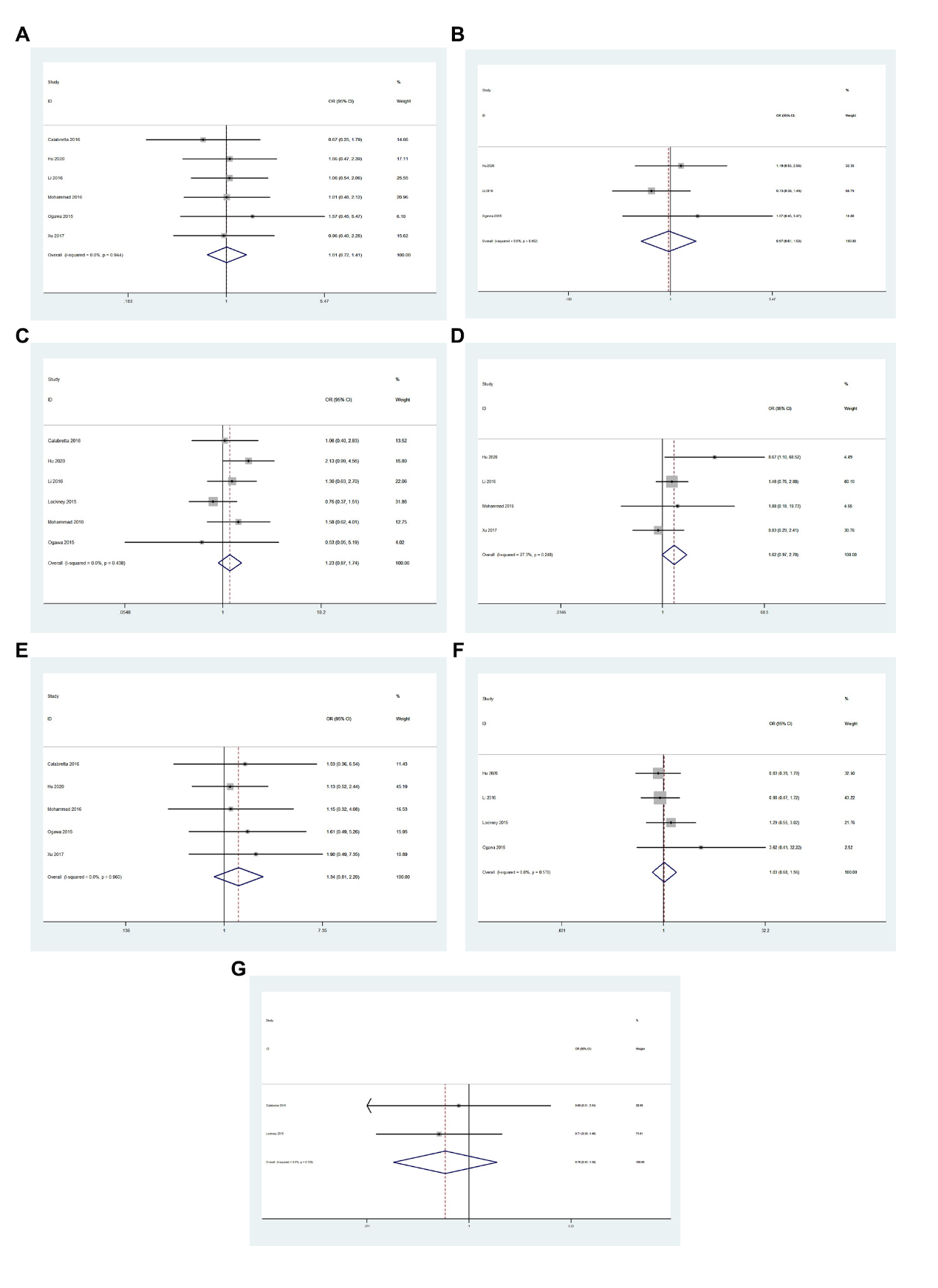


Supplementary Figure 2. Forest plots for the relationship between PKM2 and clinicopathologic characteristics in pancreatic cancer. (A) Gender; (B) Tumor location; (C) Tumor differentiation; (D) TNM stage; (E) Lymph node metastasis; (F) Perineural invasion; (G) Resection margin

**Supplementary Table 1. Quality assessment and overall risk of bias of included studies using Newcastle-Ottawa Scale.**

| First author / year | Patient selection | | | |  | Comparability |  | Outcome | | | Quality score |
| --- | --- | --- | --- | --- | --- | --- | --- | --- | --- | --- | --- |
|  | Representation of the exposed cohort | Selection of the non-exposed cohort | Ascertainment of exposure | Outcome of  interest not  present at start |  | Comparability of cohorts on the basis of the design or analysis |  | Assessment  of outcome | Was follow-up long enough for outcomes to occur | Adequacy of follow up of cohorts |  |
| Yang 2016 (20) | ☆ | ★ | ★ | ★ |  | ★★ |  | ★ | ★ | ★ | 8 |
| Takahashi 2020 (14) | ☆ | ★ | ★ | ★ |  | ★★ |  | ★ | ☆ | ☆ | 6 |
| Boira 2020 (15) | ☆ | ★ | ★ | ★ |  | ★★ |  | ★ | ★ | ★ | 8 |
| Lu 2016 (16) | ☆ | ★ | ★ | ★ |  | ★★ |  | ★ | ☆ | ★ | 7 |
| Lyshchik 2007 (21) | ☆ | ★ | ★ | ★ |  | ★ |  | ★ | ☆ | ★ | 6 |
| Pizzi 2009 (17) | ☆ | ★ | ★ | ★ |  | ★★ |  | ★ | ★ | ★ | 8 |
| Chikamoto 2017 (18) | ☆ | ★ | ★ | ★ |  | ★★ |  | ★ | ★ | ★ | 8 |
| Kitasato 2014 (19) | ☆ | ★ | ★ | ★ |  | ★ |  | ★ | ☆ | ★ | 6 |
| Baek 2014 (24) | ☆ | ★ | ★ | ★ |  | ★ |  | ★ | ☆ | ★ | 6 |
| Ogawa 2015 (25) | ☆ | ★ | ★ | ★ |  | ★★ |  | ★ | ☆ | ★ | 7 |
| Mohammad 2016 (26) | ☆ | ★ | ★ | ★ |  | ★★ |  | ★ | ★ | ★ | 8 |
| Calabretta 2016 (27) | ☆ | ★ | ★ | ★ |  | ★★ |  | ★ | ☆ | ★ | 7 |
| Xu 2017 (28) | ☆ | ★ | ★ | ★ |  | ★ |  | ★ | ☆ | ★ | 6 |
| Li 2016 (29) | ☆ | ★ | ★ | ★ |  | ★ |  | ★ | ★ | ★ | 7 |
| Lockney 2015 (30) | ☆ | ★ | ★ | ★ |  | ★★ |  | ★ | ☆ | ★ | 7 |
| Hu 2020 (31) | ☆ | ★ | ★ | ★ |  | ★★ |  | ★ | ★ | ★ | 8 |
| Wang 2019 (32) | ☆ | ★ | ★ | ★ |  | ★ |  | ★ | ☆ | ★ | 6 |
| Sun 2017 (33) | ☆ | ★ | ★ | ★ |  | ★ |  | ★ | ★ | ★ | 7 |
